# Perception, knowledge, and influencing factors of acute skin failure among critical care medical staff: a multicenter, cross-sectional study

**DOI:** 10.3389/fmed.2026.1746416

**Published:** 2026-02-05

**Authors:** Xiuhuan Huang, Mingming Zhang, Yajuan Chen, Aiwen Yang, Yeling Wu, Qiuni Cai

**Affiliations:** 1 Department of Hepatobiliary Surgery, Zhongshan Hospital of Xiamen University, School of Medicine, Xiamen University, Xiamen, China; 2 Department of General Surgery, Zhongshan Hospital of Xiamen University, School of Medicine, Xiamen, China; 3 Department of Vascular Surgery, Zhongshan Hospital of Xiamen University, School of Medicine, Xiamen University, Xiamen, China

**Keywords:** acute skin failure, critical care, intensive care units, medical staff, perception

## Abstract

**Objective:**

This study aimed to assess the knowledge level of acute skin failure (ASF) among critical care medical staff and to identify the factors influencing this knowledge.

**Methods:**

A multicenter, cross-sectional study was conducted in the intensive care units of four tertiary hospitals in Xiamen, China. A total of 481 physicians and nurses completed a validated, self-designed questionnaire assessing knowledge across eight domains of ASF.

**Results:**

The survey revealed a significant knowledge gap, with 60.5% (291/481) of participants demonstrating insufficient knowledge of ASF. Furthermore, 56.1% of respondents could not accurately differentiate ASF from pressure injuries. Multivariate logistic regression identified job category and years of experience as independent predictors of knowledge level. Physicians were significantly less knowledgeable than nurses (*OR* = 2.888, *p* = 0.017). Contrary to expectations, staff with more than 10 years of experience were less knowledgeable than their junior counterparts.

**Conclusion:**

Variations in familiarity with ASF concepts exist among critical care staff, reflecting differences in professional training frameworks, clinical responsibilities, and exposure patterns. The observed differences between physicians and nurses likely represent systematic variations in clinical focus and role-specific knowledge acquisition rather than absolute knowledge deficits. These findings highlight the need for interprofessional educational strategies that respect professional boundaries while fostering collaborative recognition of ASF as a shared clinical concern.

## 1 Introduction

Critically ill patients represent a uniquely vulnerable population, exposed to a multitude of intrinsic and extrinsic factors that predispose them to skin breakdown. These include hemodynamic instability, hypoperfusion, prolonged immobility, edema, and the use of vasopressors and medical devices (1, 2). For decades, the paradigm for skin breakdown has been centered on the prevention of pressure injuries, with their occurrence often viewed as an indicator of care quality. However, a growing body of evidence and clinical consensus suggests that not all skin breakdown in this fragile population is preventable (3, 4). This has led to the emergence and gradual acceptance of the concept of acute skin failure.

Acute skin failure (ASF) is defined as the sudden, non-pressure-related breakdown of skin attributable to hypoperfusion, inflammation, or multisystem organ failure—has only recently gained recognition as a distinct clinical entity (5, 6). Unlike chronic or mechanically induced skin lesions, ASF represents an end-organ manifestation of critical illness, often emerging rapidly despite optimal preventive measures, and portending poor prognosis (7).

Despite its clinical significance, ASF remains underrecognized and inconsistently documented in medical records. A growing body of evidence suggests that healthcare providers, including nurses and physicians, frequently misattribute ASF lesions to pressure injury or incontinence-associated dermatitis, thereby delaying appropriate interventions and compromising patient outcomes (8, 9). This diagnostic ambiguity stems, in part, from the absence of universally accepted diagnostic criteria and standardized terminology, which hinders both clinical management and research comparability (10).

Moreover, while several consensus statements and expert reviews have attempted to define ASF and propose pathophysiological mechanisms—including microcirculatory dysfunction, cytokine-mediated tissue damage, and metabolic derangements—there remains a paucity of empirical data regarding frontline medical staff’s perception, understanding, and documentation practices surrounding ASF (11, 12). Understanding these perceptual gaps is essential to developing targeted educational initiatives, refining clinical guidelines, and improving interprofessional communication around this emergent syndrome.

This cross-sectional study aims to investigate the knowledge, attitudes, and perceptions of physicians and nurses working in critical care settings regarding the concept of skin failure in critically ill patients. We hypothesize that there is significant variability and potential knowledge gaps in the recognition and understanding of skin failure among medical staff. Through this cross-sectional survey, we seek to elucidate the current state of awareness and identify factors associated with a more accurate conceptualization of this condition. The findings of this research will provide a foundational evidence base for future interventions designed to support clinicians and improve the overall approach to skin integrity at the end of life.

## 2 Methods

### 2.1 Study design and setting

A multicenter, cross-sectional study was conducted between January and February 2025 in the intensive care units of four major tertiary hospitals in Xiamen, China. This design was employed to efficiently evaluate the current state of knowledge and perceptions regarding ASF among critical care medical staff across different institutions. The study adhered to the Strengthening the Reporting of Observational Studies in Epidemiology (STROBE) guidelines for cross-sectional studies (13) (Supplementary material 1).

### 2.2 Participants and sampling

The target population comprised physicians and nurses who were directly involved in patient care within the participating ICUs.

Inclusion criteria were: (1) Registered physicians or nurses with a valid national professional qualification certificate; (2) current employment in the ICU of one of the four tertiary hospitals; (3) voluntary participation with written informed consent (implied via questionnaire submission).

Exclusion criteria were: (1) Interns, postgraduate students, or rotating staff from non-ICU departments; (2) ICU work experience <3 months.

A convenience sampling method was used to recruit participants. The sample size was calculated based on the preliminary survey results and the number of eligible medical staff across the four ICUs. To ensure a high response rate, the head nurse of each unit distributed the questionnaire link via a departmental communication platform, with two weekly reminders sent during the data collection period. A total of 500 questionnaires were distributed. After excluding 19 invalid responses (due to incompleteness or patterned answering), 481 valid questionnaires were included in the final analysis, resulting in an effective response rate of 96.2%.

### 2.3 Sample size calculation

The sample size was calculated using the formula for estimating a single population proportion: *n* = *Z*^2^ × *P*(1 − *P*)/*d*^2^, where n is the required sample size, *Z* is the Z-score corresponding to the desired confidence level (1.96 for 95% CI), *P* is the estimated proportion, and d is the margin of error.

Based on a preliminary survey and a review of similar literature (14), we anticipated that approximately 50% of medical staff would have insufficient knowledge of ASF. This proportion (*p* = 0.5) was chosen as it yields the most conservative (largest) sample size. Setting the margin of error (d) at 0.05 and the confidence level at 95%, the initial calculated sample size was *n* = (1.96)^2^ × 0.5(1–0.5)/(0.05)^2^ = 384. Considering a potential non-response rate of 20%, the final target sample size was adjusted to 460 participants. A total of 500 questionnaires were distributed to ensure an adequate number of valid responses, and 481 were included in the final analysis, which exceeded the minimum requirement.

### 2.4 Ethical considerations

The study protocol was reviewed and approved by the Human Ethics Committee of Zhongshan Hospital, Xiamen University (Approval No: xmzsyyky2023-183). The first page of the electronic questionnaire detailed the study’s purpose, procedures, and the anonymous and voluntary nature of participation. Submission of the completed questionnaire was considered implied consent from the participants.

### 2.5 Instrument development and data collection

The research instrument was a self-administered, electronic questionnaire developed through a rigorous process:

Literature Review and Item Generation: An extensive review of current literature on ASF and pressure injuries in critical care informed the initial draft of the questionnaire.

Expert Validation: A panel of five experts, including intensivists, wound care nurse specialists, and a clinical methodology, assessed the questionnaire for content validity, relevance, and clarity. The Scale-Level Content Validity Index (S-CVI) was 0.944.

Pilot Study and Reliability: A pilot test was conducted with 30 ICU staff (not included in the final sample) to check for comprehension, timing, and internal consistency. The knowledge scale demonstrated excellent reliability with a Cronbach’s *α* of 0.982.

The final questionnaire consisted of three sections:

Section A: Demographic and Professional Characteristics. This section collected information on gender, age, job category (physician/nurse), specific ICU type (e.g., medical, surgical, general, others), professional title, education level, and working years.

Section B: Knowledge of Acute Skin Failure. This section contained 10 items assessing knowledge across eight domains: definition, classification, causes, timing, diagnostic criteria, risk factors, pathophysiology, and susceptible regions of ASF. Each item was rated on a 5-point Likert scale from 1 (“very ignorant”) to 5 (“very aware”). The total possible score ranged from 8 to 40 points. A cut-off score of ≥24 (representing 60% of the total score) was pre-defined to classify participants into the “sufficient knowledge” group, while scores <24 indicated “insufficient knowledge.” The cutoff score of ≥24 (60% of total score) was established based on similar knowledge-attitude-practice (KAP) studies in clinical education, where a score above 60% is commonly used to denote “adequate knowledge” in the absence of a gold standard. This threshold was also reviewed and agreed upon by the expert panel during instrument validation.

Section C: Differentiation and Learning Willingness. This section included one open-ended question asking participants to describe the differences between skin failure and pressure injuries. It also contained a single multiple-choice question (“Are you willing to learn about acute skin failure?”) with “Yes” or “No” as options.

A unified electronic questionnaire link and QR code were distributed to the head nurses of each participating ICU. They were requested to forward the survey to all eligible physicians and nurses within their units. The survey platform was configured to ensure anonymity, prevent duplicate submissions from the same IP address, and mandate responses to all items to minimize missing data. Data collection was closely monitored by the principal investigator at each site to ensure a high response rate.

### 2.6 Statistical analysis

All statistical analyses were performed using IBM SPSS Statistics version 25.0 (IBM Corp., Armonk, NY, USA). Descriptive statistics were used to summarize participant characteristics and knowledge scores, presented as frequencies (percentages) for categorical variables and medians (interquartile ranges) for continuous variables, as the data were not normally distributed.

The Mann–Whitney U test was used to compare knowledge scores between groups for continuous variables (e.g., working years). The Chi-square test or Fisher’s exact test (where cell counts were <5) was used for univariate analysis to identify associations between categorical demographic factors and the dichotomized knowledge level (sufficient vs. insufficient).

Variables that showed a significant association (*p* < 0.05) in the univariate analysis were entered into a binary logistic regression model to identify independent factors associated with sufficient knowledge of ASF. The results were expressed as odds ratios (OR) with 95% confidence intervals (CI). A two-tailed *p*-value of < 0.05 was considered statistically significant for all tests.

## 3 Results

### 3.1 Participant characteristics

A total of 481 critical care medical staff from four hospitals completed the survey and were included in the final analysis. The demographic and professional characteristics of the participants are summarized in Table 1. The majority of respondents were female (87.5%, *n* = 421) and nurses (91.7%, *n* = 441). The largest proportion of participants were aged between 31 and 40 years (46.2%, *n* = 222), held a primary professional title (46.8%, *n* = 225), and possessed a bachelor’s degree (58.2%, *n* = 280). Participants were recruited from Medical (39.5%, *n* = 190), Surgical (26.2%, *n* = 126), General (20.8%, *n* = 100), and other specialized ICUs (13.5%, *n* = 65). Regarding working years, 42.4% (*n* = 204) had worked for more than 10 years.

**Table 1:** Demographic characteristics of participants.

Variables		Numbers of patients
Gender	Male	60 (12.5)
	Female	421 (87.5)
Age, years	<26	67 (13.9)
	26 ~ 30	94 (19.5)
	31 ~ 40	222 (45.2)
	>40	98 (20.4)
Job category	Doctor	40 (8.3)
	Nurse	441 (91.7)
Professional title	Primary title	225 (46.8)
	Intermediate	214 (44.5)
	Senior	42 (8.7)
Educational level	Below bachelor’s	177 (36.8)
	Bachelor’s	280 (58.2)
	Master’s or higher	24 (5.0)
ICU type	Medical ICUs	190 (39.5)
	Surgical ICUs	126 (26.2)
	General ICUs	100 (20.8)
	Others	65 (13.5)
Working years	<6 y	149 (31.0)
	6 ~ 10 y	128 (26.6)
	>10 y	204 (42.4)

### 3.2 Knowledge level of acute skin failure

The overall knowledge level of ASF among the medical staff was low. As detailed in Table 2, the proportion of participants who reported being “well acquainted” or “very well acquainted” with the various domains of ASF was below 20% for all items. The highest rate of familiarity was observed for the ‘susceptible regions of ASF’ (24.3%, 117/481), while the lowest was for the ‘diagnostic criteria of ASF’ (16.4%, 79/481).

**Table 2:** Medical staff’s perception of ASF [n(%)].

Content	Option (*n* = 481)				
	Very ignorant (1 point)	Not acquainted (2 points)	Average (3 points)	Well acquainted (4 points)	Very well acquainted (5 points)
Definition of ASF	80 (16.7)	144 (29.9)	150 (31.2)	90 (18.7)	17 (3.5)
Classification of ASF	104 (21.6)	164 (34.1)	127 (26.4)	70 (14.6)	16 (3.3)
Causes of ASF	92 (19.1)	129 (26.8)	164 (34.1)	83 (17.3)	13 (2.7)
Timing of ASF	100 (20.8)	138 (28.7)	152 (31.6)	77 (16.0)	14 (2.9)
Diagnostic criteria of ASF	113 (23.5)	159 (33.1)	130 (27.0)	63 (13.1)	16 (3.3)
Risk factors of ASF	98 (20.4)	135 (28.1)	150 (31.2)	80 (16.6)	18 (3.7)
Pathophysiology of ASF	107 (22.2)	147 (30.6)	147 (30.6)	65 (13.5)	15 (3.1)
Susceptible regions of ASF	94 (19.5)	123 (25.6)	147 (30.6)	93 (19.3)	24 (5.0)

Based on the pre-defined cut-off score (≥24 indicating sufficient knowledge), 39.5% (*n* = 190) of the medical staff were classified into the sufficient knowledge group, while 60.5% (*n* = 291) were categorized into the insufficient knowledge group.

### 3.3 Factors associated with ASF knowledge: univariate analysis

Univariate analyses were performed to identify factors associated with the level of ASF knowledge (sufficient vs. insufficient). The results are presented in Table 3.

**Table 3:** Univariate analysis of medical staff’s perception of skin failure in critically ill patients (*n* = 481).

Variable		N[n(%)]	Scoring ≥24 (*n* = 190)	Scoring < 24 (*n* = 291)	*χ*^2^ value	*P*-value
Gender	Male	60 (12.5)	22 (36.7)	38 (63.3)	0.230	0.631
	Female	421 (87.5)	168 (39.9)	253 (60.1)		
Age, years	<26	67 (13.9)	35 (52.2)	32 (47.8)	11.798	0.008
	26 ~ 30	94 (19.5)	37 (39.4)	57 (60.6)		
	31 ~ 40	222 (46.2)	92 (41.4)	130 (58.6)		
	>40	98 (20.4)	26 (26.5)	72 (73.5)		
Job category	Doctor	40 (8.3)	7 (17.5)	33 (82.5)	8.837	0.001
	Nurse	441 (91.7)	183 (41.5)	258 (58.5)		
Professional title	primary	225 (46.8)	100 (44.4)	125 (55.6)	4.695	0.096
	intermediate	214 (44.5)	77 (36.0)	137 (64.0)		
	senior	42 (8.7)	13 (31.0)	29 (69.0)		
Educational level	Below bachelor’s	177 (36.8)	82 (46.3)	95 (53.7)	5.902	0.052
	Bachelor’s	280 (58.2)	101 (36.1)	179 (63.9)		
	Master’s or higher	24 (5.0)	7 (29.2)	17 (70.8)		
ICU type	Medical ICUs	190 (39.5)	73 (38.4)	117 (61.6)	14.224	0.009
	Surgical ICUs	126 (26.2)	42 (33.3)	84 (66.7)		
	General ICUs	100 (20.8)	55 (55.0)	45 (45.0)		
	Others	65 (13.5)	20 (30.8)	45 (69.2)		
Working years	<6 y	149 (31.0)	68 (45.6)	81 (54.4)	11.017	0.04
	6 ~ 10 y	128 (26.6)	59 (46.1)	69 (53.9)		
	≥10 y	204 (42.4)	63 (30.9)	141 (69.1)		

Significant associations were found between knowledge level and several demographic factors. Specifically, a higher proportion of staff with sufficient knowledge were found in younger age groups (*p* = 0.008). Nurses demonstrated a significantly higher rate of sufficient knowledge (41.5%, 183/441) compared to physicians (17.5%, 7/40) (*p* = 0.001). The distribution of knowledge levels also varied significantly across different ICU types (*p* = 0.009), with staff in General ICUs showing the highest proportion of sufficient knowledge (55.0%, 55/100). Furthermore, a significant association was observed with years of working experience (*p* = 0.04), wherein staff with fewer than 10 years of experience exhibited a higher rate of sufficient knowledge.

No statistically significant associations were found between knowledge level and gender, professional title, or educational level (all *p* > 0.05).

### 3.4 Independent factors influencing ASF knowledge: multivariate logistic regression

Variables that showed significance in the univariate analysis (age, job category, ICU type, working years) were included in a binary logistic regression model to identify independent factors associated with sufficient knowledge of ASF. The assignments of independent variables are shown in Table 4.

**Table 4:** The assignments of the independent variables.

Independent variables	Assignments
Age, years	<26 = 1; 26 ~ 30 = 2; 31 ~ 40 = 3; >40 = 4
Job category	Doctor = 1; Nurse = 2
ICU type	Medical ICUs = 1; Surgical ICUs = 2; General ICUs = 3; Others = 4
Working years	<6 years = 1; 6 ~ 10 years = 2; ≥ 10 years = 3

The logistic regression analysis (Table 5) revealed that job category and years of working experience were independent predictors of ASF knowledge.

**Table 5:** Related factors of medical staff’s cognition of skin failure: logistic regression results (*n* = 481).

Variable	*β*	S.E.	Wald	*P*	OR	95% Cl	
						Lower limit	Upper limit
Age, y							
>40			5.853	0.119			
<26	−0.464	0.520	0.795	0.372	0.629	0.227	1.742
26 ~ 30	0.099	0.447	0.049	0.825	1.104	0.459	2.654
31 ~ 40	−0.418	0.284	2.164	0.141	0.658	0.377	1.149
Job category							
Nurse							
Doctor	1.060	0.446	5.663	0.017	2.888	1.206	6.916
Department							
Others			7.037	0.071			
Medical ICUs	−0.181	0.321	0.319	0.572	0.834	0.444	1.565
Surgical ICUs	0.048	0.345	0.019	0.890	1.049	0.533	2.064
General ICUs	−0.671	0.361	3.459	0.063	0.511	0.252	1.037
Working years							
≥10 years			5.682	0.048			
<6 years	−0.657	0.211	9.663	0.002	1.929	1.275	2.918
6 ~ 10 years	−0.606	0.255	5.633	0.018	0.545	0.331	0.900
Constant	1.140	0.338	11.399	0.001	3.127		

Job Category: Physicians were significantly less likely to have sufficient knowledge of ASF compared to nurses (OR = 2.888, 95% CI: 1.206–6.916, *p* = 0.017).

Working Years: Staff with more than 10 years of experience were used as the reference category. Staff with less than 6 years of experience were significantly more likely to have sufficient knowledge (OR = 1.929, 95% CI: 1.275–2.918, *p* = 0.002). Similarly, staff with 6–10 years of experience also showed a higher likelihood of sufficient knowledge compared to the reference group (OR = 0.545, 95% CI: 0.331–0.900, *p* = 0.018), though the interpretation of the OR relative to the reference group should be noted.

Age and ICU type, which were significant in univariate analysis, were not retained as independent significant factors in the final multivariate model (*p* > 0.05).

## 4 Discussion

This multicenter, cross-sectional study provides a systematic investigation into the knowledge and perceptions of acute skin failure among critical care medical staff in China. The principal findings reveal a concerning deficit in ASF knowledge, with only 39.5% of participants demonstrating sufficient understanding. Furthermore, our analysis identified professional role and clinical experience as independent predictors of knowledge level, highlighting specific demographic profiles that may benefit from targeted educational interventions.

The most striking finding of this study is the profound gap in ASF knowledge, with over 60% of ICU physicians and nurses possessing insufficient knowledge. This pervasive lack of awareness aligns with the conclusions of qualitative studies and systematic reviews, which have consistently pointed to ASF being an under-recognized and poorly understood clinical entity (10, 11). For instance, Dalgleish et al., in their systematic review, noted a significant “knowledge-to-practice gap” and a scarcity of high-quality evidence to guide clinicians, which resonates with our quantitative findings (15). However, our study moves beyond merely identifying the existence of a problem by quantifying its scale in a multicenter setting, providing a robust baseline from which to measure the effectiveness of future interventions. The particularly low familiarity with diagnostic criteria (16.4%) underscores a critical barrier to accurate clinical identification, potentially explaining why ASF lesions are frequently misclassified as preventable pressure injuries (16, 17). This diagnostic inaccuracy can lead to inappropriate benchmarking, undue blame placed on nursing staff, and a failure to set realistic patient and family expectations regarding the trajectory of critical illness (10).

A pivotal finding of our regression analysis is that physicians demonstrated lower familiarity with ASF concepts compared to nurses (OR = 2.888, *p* = 0.017). This difference must be interpreted within the context of professional training paradigms, scope of practice limitations, and routine clinical responsibilities. Nursing education and practice frameworks place significant emphasis on comprehensive skin assessment as a core competency and primary responsibility. National nursing standards and professional guidelines explicitly include skin integrity monitoring as a fundamental aspect of patient assessment and care planning (18, 19). Conversely, physician training in critical care predominantly focuses on systemic organ support and life-threatening conditions—cardiovascular instability, respiratory failure, and multi-organ dysfunction—with less emphasis on dermatological manifestations unless they signal broader physiological deterioration (20, 21). Importantly, this observed knowledge difference may be partially influenced by measurement bias inherent in our assessment instrument. The questionnaire items predominantly assessed knowledge domains that align with nursing practice responsibilities rather than physician priorities in critical care settings (22). Without accounting for this role-specific exposure bias, the measured knowledge gap may overstate actual differences in clinical understanding of ASF as a concept. This finding is consistent with Mileski et al., who observed that “ownership” of skin assessment is predominantly assigned to nursing staff within interprofessional teams, creating a structural division of responsibilities that shapes knowledge acquisition patterns (23). Our results highlight the need for interprofessional educational initiatives that acknowledge these contextual differences—integrating ASF concepts into critical care medicine curricula while respecting professional boundaries and leveraging complementary expertise.

The observed differences in ASF knowledge must be carefully interpreted within distinct professional frameworks. Nurses’ daily responsibilities include routine, comprehensive skin assessments as mandated by nursing standards and protocols, creating frequent exposure to skin integrity concepts. Physicians, while responsible for holistic patient management, typically encounter ASF during acute deterioration events or complication management rather than during routine monitoring (24). This differential exposure pattern shapes knowledge acquisition trajectories. Therefore, the measured knowledge disparity likely reflects systematic differences in clinical focus, task allocation, and professional socialization rather than inherent competence gaps. Future educational interventions should be tailored to these distinct professional contexts while fostering collaborative recognition of ASF as an interdisciplinary concern.

Contrary to the conventional assumption that knowledge accumulates with experience, our data revealed an inverse relationship. Medical staff with more than a decade of experience were significantly less knowledgeable about ASF than their junior counterparts. While the univariate analysis suggested a linear relationship, the multivariate model pinpointed that those with <6 years and 6–10 years of experience were significantly more knowledgeable than the >10 years group. This intriguing finding diverges from some studies on general critical care knowledge but may be uniquely explained by the novelty of the ASF concept. The inverse relationship between clinical experience and ASF knowledge may reflect a generational gap in medical education (25). Recent graduates are more likely to have been exposed to emerging concepts such as ASF in their formal training or through updated clinical guidelines. In contrast, senior clinicians may rely on entrenched diagnostic schemas, where skin breakdown is habitually classified as pressure injury (26). This highlights the need for lifelong learning initiatives and updates in continuing medical education, particularly in rapidly evolving areas like wound care (27).

The original explanation attributing this solely to a decline in empathy and job burnout, while plausible, might be an oversimplification (28–30). A more nuanced interpretation, supported by adult learning theory, is that recent graduates have been exposed to the evolving concept of ASF during their formal education, which has only recently entered academic and professional discourse (1). In contrast, senior staff, whose foundational training may have occurred decades ago, might not have encountered ASF unless they proactively engage in continuous professional development focused on wound care. This is compounded by the “ceiling effect” of experience, where seasoned clinicians may become entrenched in established diagnostic patterns (e.g., labeling all skin breakdown as pressure injury) and demonstrate less cognitive flexibility in integrating new paradigms (31). Therefore, the issue may be less about a lack of empathy and more about the rapid evolution of medical knowledge and the challenges of lifelong learning, particularly for a concept that crosses traditional disciplinary boundaries.

The inability of 56.1% of staff to differentiate ASF from pressure injuries is a central finding that carries direct clinical implications. This confusion can trigger a cascade of adverse outcomes: misallocation of resources toward ineffective pressure-relieving strategies alone, inaccurate reporting that skews quality metrics, and potential legal ramifications when unavoidable skin failure is incorrectly documented as a preventable adverse event (32). Our results empirically confirm the conceptual ambiguity described in consensus statements and reviews (33). However, the strong expressed willingness to learn (90.4%) represents a powerful and actionable opportunity. It indicates that the knowledge gap is not due to apathy but rather to a lack of accessible, structured education. This high motivation provides a compelling mandate for healthcare institutions to develop and implement standardized training modules, decision-support tools, and clear clinical guidelines that empower staff to make accurate distinctions.

### 4.1 Implications for clinical practice and research

Based on the findings of this study, we propose the following recommendations to advance both clinical practice and research on ASF. First, it is essential to develop standardized interprofessional education programs that deliver targeted training for both nurses and—crucially—physicians, focusing on the pathophysiology, diagnosis, and clinical management of ASF. Furthermore, validated screening tools or checklists should be integrated into electronic health records to support consistent and accurate identification of ASF in clinical settings. This approach should be complemented by the active promotion of a “Just Culture” within healthcare institutions—an environment that clearly differentiates between preventable harm and unavoidable physiological events, thereby mitigating blame and reducing moral distress among healthcare staff when ASF occurs. Finally, future research should employ qualitative inquiry to explore the specific barriers and facilitators affecting ASF knowledge acquisition among senior clinicians and physicians, while multicenter studies conducted across diverse healthcare systems are needed to elucidate the influence of cultural and systemic factors on the perception and management of ASF.

### 4.2 Limitations

This study also has the following limitations. First, the cross-sectional design precludes causal inference. Second, although the questionnaire was rigorously validated, its content was primarily constructed based on nursing and wound care literature, which may align more closely with the daily work scenarios and knowledge frameworks of nurses. Consequently, the measurement may inadvertently favor the nursing group. This ‘role-related measurement bias’ could partially explain the observed differences in knowledge between physicians and nurses, and the results should be interpreted with caution. Third, convenience sampling could introduce selection bias, and the uneven sample distribution (91.7% nurses vs. 8.3% physicians) limits the generalizability of interprofessional comparisons, as the small physician subgroup may not adequately represent their broader knowledge base. Additionally, the sample was recruited from four hospitals within a single city, which may restrict the extrapolation of findings to other regions or healthcare settings. Future studies should aim for more balanced recruitment across professional roles to strengthen role-based comparisons, adopt longitudinal designs to assess knowledge retention after interventions, and validate the survey instrument in more diverse populations.

## 5 Conclusion

In conclusion, this multicenter study identifies significant variations in familiarity with Acute Skin Failure concepts among critical care medical staff, with knowledge levels closely tied to professional role and clinical exposure patterns. The observed differences between physicians and nurses reflect systematic variations in clinical responsibilities, educational emphasis, and measurement approaches rather than absolute knowledge deficits. These findings highlight the need for role-specific educational strategies that respect professional boundaries while fostering collaborative recognition of ASF. Future initiatives should focus on developing interprofessional frameworks that leverage complementary expertise to improve ASF identification and management in critically ill patients.

## Data Availability

The original contributions presented in the study are included in the article/Supplementary material, further inquiries can be directed to the corresponding author/s.
